# Depression treatment and short-term healthcare expenditures among elderly Medicare beneficiaries with chronic physical conditions

**DOI:** 10.1186/1477-5751-12-15

**Published:** 2013-10-22

**Authors:** Chan Shen, Neel Shah, Patricia A Findley, Usha Sambamoorthi

**Affiliations:** 1 Department of Biostatistics, Division of Quantitative Sciences, The University of Texas MD Anderson Cancer Center, Houston, TX, USA; 2 Department of Pharmaceutical Systems and Policy, School of Pharmacy, West Virginia University, PO Box 9510, Morgantown, WV 26506-9510, USA; 3 School of Social Work, Rutgers University, 536 George Street, New Brunswick, NJ 08901, USA

**Keywords:** Chronic physical conditions, Depression, Antidepressants, Expenditures

## Abstract

**Background:**

Research on the impact of depression treatment on expenditures is nascent and shows results that vary from negative associations with healthcare expenditures to increased expenditures. However many of these studies did not include psychotherapy as part of the depression treatment. None of these studies included “no treatment” as a comparison group. In addition, no study has included a broad group of chronic physical conditions in studying depression treatment expenditures.

**Objective:**

We determined the association between depression treatment and short-term healthcare expenditures using a nationally representative sample of Medicare beneficiaries with chronic physical conditions and depression.

**Method:**

In this retrospective cohort study, we examined the association between depression treatment in the baseline year and healthcare expenditures in the following year using data from 2000 through 2005 of the Medicare Current Beneficiary Survey (MCBS), a nationally representative survey of Medicare beneficiaries. Using the rotating panel design of MCBS, we derived five two-year cohorts: 2000–2001, 2001–2002, 2002–2003, 2003–2004, and 2004–2005. The study sample included 1,055 elderly Medicare beneficiaries aged 65 or over. We compared healthcare expenditures of no depression treatment group with depression treatment groups using t-tests. Linear regressions of log-transformed dollars were used to assess the relationship between depression treatment and healthcare expenditures after controlling for demographic, socio-economic, health status, lifestyle risk factors, year of observation and baseline expenditures.

**Results:**

Compared to no depression treatment ($16,795), the average total expenditures were higher for those who used antidepressants only ($17,425) and those who used psychotherapy with or without antidepressants ($19,733). After controlling for the independent variables, antidepressant use and psychotherapy with or without antidepressants were associated with 20.2% (95% CI: 14.1-26.7%) and 29.4% (95% CI: 18.8-41.0%) increase in total expenditures, respectively. We observed that depression treatment was positively associated with inpatient, medical provider and prescription drug expenditures.

**Conclusion:**

Among the elderly Medicare beneficiaries with chronic physical conditions, depression treatment was associated with greater short-term healthcare expenditures. Future research needs to replicate these findings and also examine whether depression treatment reduces expenditures over a longer period of time.

## Introduction

Research on how depression treatment influences healthcare expenditures among individuals with co-occurring depression and chronic physical conditions such as diabetes, heart disease, and others is an emerging area of research. In many of these chronic conditions, depression is associated with higher expenditures and treating depression may be one pathway to reduced healthcare expenditures with chronic physical conditions and depression [1]. However, findings from studies that examined the association between depression treatment and healthcare expenditures have produced mixed results. Both negative and positive associations between depression treatment and healthcare expenditures have been reported [2]. Among adults with diabetes, a randomized control trial was conducted to compare systematic depression treatment program to collaborative care with usual care. Findings from this trial revealed that systematic depression treatment significantly increased time free of depression and resulted in economic benefits from the insurer’s perspective [3]. In a single disease framework, among veterans with diabetes, depression treatment was associated with reduced total healthcare expenditures [4]. A study on collaborative care for individuals with depression and poorly controlled chronic physical conditions such as diabetes and/or coronary heart disease concluded that collaborative care improved chronic care outcomes such as glycated hemoglobin, low-density lipoprotein (LDL) cholesterol, and systolic blood-pressure levels in these patients [5]. These findings suggest that depression treatment may reduce healthcare expenditures through improvement in chronic care outcomes or reduction of depressive symptoms.

Other studies found increased healthcare expenditures with depression treatment [6]. A stepped collaborative care program for individuals with depression in primary care settings resulted in substantial increases in treatment effectiveness defined as depression free days and moderate increases in expenditures [6]. A systematic review of randomized economic evaluations concluded that although collaborative/case management approach for depression may improve outcomes, such approaches may be expected to increase cost and will require initial investment [7].

It has to be acknowledged that these studies have contributed to the emerging literature on the association between depression treatment and healthcare expenditures among individuals with chronic conditions. However many of these studies did not include psychotherapy as part of the depression treatment. None of these studies included “no treatment” as a comparison group. It is possible that depression treatment may be delayed due to either competing demands of physical conditions [8-10] or watchful waiting due to adverse effects of antidepressants on incident diabetes and heart disease [11,12] or high rates of treatment failures and lack of robust evidence of depression treatment on chronic care outcomes [13,14]. In addition, the focus of these studies were on evaluating specific programs of collaborative care. However, many individuals seek care in usual-care settings and an evaluation of depression treatment on healthcare expenditures in real-world practice settings is needed.

Therefore, the primary objective of the study is to analyze the association between depression treatment and healthcare expenditures among individuals with chronic conditions and seeking care in real-world practice settings. Based on evidence from existing studies, we hypothesize that there is a positive association between depression treatment and short-term expenditures among individuals with chronic conditions. The current study makes a unique contribution by including a broad group of chronic physical conditions, analyzing the major modalities of depression treatment: antidepressant use and psychotherapy, and including a group of individuals who did not receive depression treatment. Our study findings can inform the new emerging policy area of comparative effectiveness which focuses on comparing alternative methods of treatment in real world settings to improve both individual and population health. For purposes of the current study we used longitudinal data on a nationally representative sample of Medicare beneficiaries with chronic physical conditions.

## Methods

### Conceptual framework

The current paper examines the association between depression treatment and healthcare expenditures. It is plausible to have a host of factors that are associated with healthcare expenditures. Therefore, to guide our selection of variables that may affect healthcare expenditures, we used the expanded behavioral model on use of health services proposed by Andersen [15]. Briefly, the model posits that utilization of health services varies as a function of 1) each individual’s unique predisposition for using services (predisposing factors), which includes gender, race, age, and observation year in our study; 2) the means available to each individual for obtaining services (enabling factors), which includes marital status, education, poverty status, and prescription drug coverage in our study; 3) each individual’s level of need, which includes perceived health status and functional status in our study; 4) personal health practices and use of health services, including smoking status, body mass index (BMI), depression treatment, and the baseline year log-transformed health expenditures in our study; and 5) the external environment, which is metro status in this study.

### Study design

This is a retrospective cohort study that examined the association between depression treatment and short-term healthcare expenditures. In our study, we captured depression treatment in the first (baseline) year; and measured short-term expenditures as healthcare expenditures in the second (follow-up) year, because antidepressants have been shown to be effective after 4–5 weeks and their effect can persist up to 18 weeks [16]. We refer to the first year as the baseline year and the second year as the follow-up year.

### Data source

To examine the direction and magnitude of association between depression treatment and healthcare expenditures, data from multiple years of the *Cost and Use files* of the Medicare Current Beneficiary Survey (MCBS) were used. The current study uses data from the Cost and Use files for years 2000 through 2005, the most recent years of observation available at the time of the study. The MCBS is an ongoing, multipurpose survey of a representative sample of the Medicare population. It is a unique and comprehensive source of information on the health status, healthcare use, health insurance coverage, and socioeconomic and demographic characteristics of Medicare beneficiaries [17]. The MCBS combines information from beneficiary interviews with data from Medicare claims. The MCBS has a rotating panel design in which survey respondents were interviewed 12 times over a period of 4 years to get complete information for 3 years [17]. We used this rotating panel design to our advantage and captured depression treatment in the baseline year and examined expenditures in the subsequent follow-up year. As a result our study includes five cohorts: 2000–2001; 2001–2002; 2002–2003; 2003–2004; and 2004–2005. Although it is possible to have repeated observations on some characteristics of the beneficiaries from different modules of the survey (e.g., race was surveyed in two modules), we included only one set of longitudinal data for each Medicare beneficiary as we expect most of the repeated information to be duplicate.

#### Identification of depression

Depression diagnosis is based on *International Classification of Diseases, 9*^*th*^*Edition, Clinical Modification* (ICD-9-CM) codes extracted from the Medicare claims. Primary and secondary diagnostic codes were used to identify beneficiaries who received a diagnosis of depression from one of their providers during a healthcare encounter. We followed HEDIS guidelines in identifying depression [18] and included the following ICD-9-CM codes: 296.2 (major depressive disorder, single episode), 296.3 (major depressive disorder, recurrent episode), 309.1 (prolonged depressive reaction), 300.4 (neurotic depression), and 311 (depression, not elsewhere classified). These codes have been used to identify diagnosed depression in prior published studies [19-22].

#### Presence of chronic physical conditions

Based on self-reports from the survey data, we defined the presence or absence of each of the following chronic physical conditions: arthritis, diabetes, respiratory diseases including asthma, chronic obstructive pulmonary disease, heart disease, hypertension, osteoporosis, and stroke. For example, based on positive response to queries on whether a doctor told the patient whether he/she had diabetes, we classified respondents as having diabetes.

#### Analytical sample

Our analytical sample was based on Medicare beneficiaries who were diagnosed with depression during the baseline year and reported any one or more of the chronic physical conditions listed in the previous paragraph. We further restricted our analytical sample to Medicare beneficiaries who were 65 and older, community-dwelling during the observation period, were followed for 3 years, and were enrolled in Medicare Fee-for-Service (FFS) plans because claims are available only for FFS beneficiaries and not for services provided through Medicare managed care plans. After all our exclusions, our final sample consisted of 1055 person years with depression and at least one chronic condition.

### Measures

#### Dependent variables: log--transformed healthcare expenditures

The MCBS combines healthcare expenditures from many sources. These include payments made by third-party payers including Medicare, Medicaid, Medicare-Health Maintenance Organizations (HMO), Veterans Affairs Health Insurance, private HMO, individually purchased insurance, employer-sponsored insurance, other payers and those paid by self or family. In addition to total healthcare, we also analyze sub-types of expenditures, which included inpatient, outpatient, medical providers, prescription, and other. Other expenditures consisted of home health, vision, dental, and durable medical equipment. In all analyses expenditures were adjusted for medical care services inflation and reported in 2005 dollars. We used the annual consumer price index for medical care services provided by the Bureau of Labor Statistics [23].

#### Key independent variable: depression treatment

##### Any antidepressant use

Indicators for each respondent were created, based on self-report, whether they had any use of antidepressants (both brands and generics) during the calendar year in which a diagnosis of depression took place. Drugs included in the class of antidepressants were amitriptyline, amoxapine, bupropion, clomipramine, desipramine, doxepin, imipramine, isocarboxazid, maprotiline, nefazodone, nortriptyline, phenelzine, protriptyline, tranylcypromine, trazodone, trimipramine, and venlafaxine, as well as the SSRIs, which included fluoxetine, fluvoxamine, paroxetine, and sertraline.

##### Psychotherapy

Use of psychotherapy was identified by using physician’s current procedure terminology (*Physician’s Current Procedural Terminology, CPT *) and Health Care Financing Administration Common Procedure Coding System (HCPCS) codes in the Medicare claims. Psychotherapy was defined broadly based on a complied list of CPT and HCPCS codes reviewed by psychologists and psychiatrists [22].

#### Other independent variables

Predisposing characteristics were: gender (women/men), race (White, African American, other), age (categorized as 65–69, 70–74, 75–79, and 80 years and over) and year of observation (2000–2001, 2001–2002, 2002–2003, 2003–2004, 2004–2005). Enabling characteristics included marital status (married, other), education (less than high school, high school, some college, college), poverty status measured as income to poverty ratio or federal poverty level (less than 200% and greater than or equal to 200%) and prescription drug coverage (yes and no). Need characteristics included two variables: perceived health status (excellent or very good, good, fair or poor) and functional status, which is the number of activities of daily living (ADLs) with limitations (none, 1–2 limitations, 3–6 limitations). Personal health practices and use of health services included smoking status (current, past, never), BMI (under weight or normal, i.e., BMI <25 kg/m^2^, overweight, i.e., BMI ≥25- 29.9 kg/m^2^, obese, i.e. BMI ≥ 30 kg/ m^2^) [24], and baseline year log-transformed health expenditures [25]. External environment was captured by metro status (metro, not metro).

The reference group for the independent variables were: gender (men), race (white), age (65–69 years), year of observation (2004–2005), marital status (married), education (college), poverty status (greater than or equal to 200%), prescription drug coverage (yes), health status (excellent or very good), functional status (no ADL limitations), smoking status (never), and BMI (obese).

### Statistical analysis

Chi-square tests were used to test unadjusted group differences in depression treatment. Chi-squares were derived from performing two-way tabulations of depression treatment and subject characteristics with surveyfreq procedures in SAS. To compare the differences in average healthcare expenditures between no depression treatment and treatment groups we used t-tests. Separate linear regression models for each type of expenditures with ordinary least squares (OLS) estimation were employed to assess the relationship between depression treatment and healthcare expenditures after controlling for predisposing, enabling, need, personal health practices and health services use, and external environment factors. In these multiple linear regressions, healthcare expenditures were transformed using natural logarithm to normalize the error distribution. Point estimates of the coefficients of continuous independent variables on the log of monthly expenditures multiplied by 100 can be interpreted as percentage change for each unit of change in the independent variable. The effect of dummy variables on healthcare expenditures in terms of percentage of expenditures can be estimated by exponentiating the regression coefficients of dummy variables subtracting one (i.e., percent change=e^β^-1) and then multiplying by 100 [26]. All tests were two-sided, and the significant level was set at 0.05.

All analyses were conducted using survey procedures (i.e., svyfreq, svymeans and svyreg) and in SAS 9.3 (SAS Institute, Cary NC) in order to account for the complex survey design of the MCBS. In these procedures domain statement or appropriate SAS syntax was used to conduct analysis on the sub-group of elderly individuals with depression. For example, the MCBS file contains weights for each of the beneficiaries in the data set. These weights reflect the overall selection probability of each sample person and include adjustment for survey nonresponse and post-stratification to control totals based on accretion status, gender, age, race, region, and metropolitan area status. In addition to the ongoing continuous sampling and rotating panel design, the MCBS oversamples persons over the age of 80 to ensure annual sample yields are enough to produce statistically reliable data. Therefore, in the current study we report weighed percentages and all our analyses appropriately used SAS survey procedures.

## Results

The description of the study sample by depression treatment categories is summarized in Table 1. Because of the complex survey design of the MCBS, we report weighted percentages in this table. Only elderly individuals who were enrolled in fee-for-service Medicare for the full year and did not die during the observation period were included. Therefore medical claims for each patient in every cohort were available for the entire 24 month period.

**Table 1:** **Description of study sample by type of depression treatment, medicare current beneficiary survey, 2000-2005**^**a**^

		**AD only**		**Psych Tx (with or w/o AD)**		**No Tx.**	
		**N**	**Wt %**	**N**	**Wt %**	**N**	**Wt %**
**Observation years**							
	2000-2001	70	42.2	34	23	60	34.7
	2001-2002	113	59.4	28	14.4	54	26.3
	2002-2003	104	48.5	41	19.9	69	31.6
	2003-2004	132	59.8	33	14.4	55	25.8
	2004-2005	137	53.3	45	18	80	28.7
**Gender**							
	Female	409	53.2	137	18.4	228	28.4
	Male	147	53.5	44	15.5	90	30.9
**Race**							
	White	487	55.2	145	16.8	258	28.0
	African American	24	39.5	12	22.5	23	37.9
	Other	45	43.5	24	23.0	37	33.4
**Age in years**							
	65-69	111	53	36	19	59	28
	70-74	119	57.4	36	17.4	55	25.1
	75-79	110	51	42	19.4	63	29.6
	80 +	216	52.1	67	15.7	141	32.2
**Marital status**							
	Married	239	55.8	68	16.6	118	27.6
	Other	317	51.5	112	18.3	200	30.2
**Metro status**							
	Metro	397	53.7	145	19.6	211	26.7
	Not Metro	159	52	36	11.9	107	36.1
**Education**							
	LT High school	176	52.7	50	14.1	121	33.2
	High school	218	53.2	58	14.9	128	31.9
	Some college	68	49.7	30	22.8	38	27.5
	College	91	57.5	43	27.3	29	15.2
**Poverty status**							
	LT 200%	314	51	95	15.7	214	33.4
	GE 200%	242	56.4	86	20.4	104	23.2
**Prescription drug coverage**							
	Yes	446	54.2	146	17.9	243	27.9
	No	110	49.4	35	16.5	75	34.1
**Perceived health status**							
	Excellent/very good	137	50.4	53	20.4	81	29.2
	Good	194	54.7	60	17.6	105	27.7
	Fair/poor	221	53.9	67	16.1	129	30.0
**Functional status (ADL)**							
	None	285	50.8	95	18.2	179	31.1
	1-2	162	54.5	53	16.8	91	28.7
	3-6	106	58.4	33	18.1	47	23.4
**Smoking status**							
	Current	57	53.2	17	18.6	31	28.2
	Past	263	54.0	82	16.6	155	29.3
	Never	235	52.8	82	18.8	128	28.4
**BMI**							
	Underweight/normal	237	52.4	84	19.5	140	28.2
	Overweight	178	52.4	54	14.9	111	32.7
	Obese	137	55.9	42	18.6	64	25.6

Note: Based on 1,055 elderly community-dwelling Medicare beneficiaries aged 65 years or older, and followed for one year during 2001 through 2005. All characteristics and depression treatment were measured in year 1.

^a^Due to small number of individuals who received only psychotherapy but no antidepressant treatment, we combined people having psychotherapy with and without antidepressant treatment into one group.

Wt.: Weighted. LT: Less than; GE: Greater than or equal to; Tx: Treatment; AD: Antidepressants; Psych Tx (with or w/o AD): Psychotherapy with or without antidepressants; ADL: Activities of daily living; BMI: Body Mass Index.

Statistically significant differences in depression treatment rates were found by race, metro status, educational level, and poverty status. A significantly lower percent of African Americans (39.5%) compared to whites (55.2%) used antidepressants only. A significantly higher percent of individuals living in metro areas (53.7%) compared to non-metro areas (52%) reported using antidepressants. Psychotherapy with or without antidepressants was more likely among individuals living in metro (19.6%) versus non metro areas (11.9%). A significantly higher proportion of college graduates (57.5%) used antidepressants compared to those with a less than high school (52.7%), high school (53.2%) and some college (49.7%) level of education. Psychotherapy was also more common among college graduates (27.3%) compared to those with an education less than high school (14.1%), high school (14.9%) or just some college (22.8%).

The average expenditures for different categories (i.e. total, inpatient, outpatient, medical provider, prescription drugs, and other) and standard deviations by depression treatment groups are presented in Table 2. The omnibus F-test showed p-values less than 0.001 with 31 degrees of freedom for all the above categories of expenditures. Overall, compared to no depression treatment ($16,795) average total expenditures were higher for those who used antidepressants only ($17,425), a difference of $630. However, this difference was not statistically significant. Compared to no depression treatment, those who used psychotherapy with or without antidepressants had higher average total expenditures ($19,733), a difference of $2,938. This difference was statistically significant (p = .005). When examined by type of expenditures, we did not observe statistically significant differences in average inpatient expenditures and outpatient expenditures by depression treatment categories (Table 2). However, for medical provider and prescription drugexpenditures individuals who used psychotherapy with or without antidepressants had the highest expenditures followed by those who used antidepressants only for depression treatment, and those without any treatment for depression. For example, the average prescriptions drug expenditures (i.e. all drugs including antidepressants) were $3,076, $2,848, and $1829 respectively.

**Table 2:** **Weighted average expenditures and standard deviations by type of depression treatment and gender, medicare current beneficiary survey, 2000-2005**^**a**^

**Expenditures**	**Antidepressants only**			**Psych Tx (with or w/o AD)**			**No treatment**	
	**Mean $**	**SD $**	***p-value***	**Mean $**	**SD $**	***p-value***	**Mean $**	**SD $**
**Total**	17,425	18,038.4	0.296	19,733	18,448.7	0.005	16,795	17,956.7
**Inpatient**	5,186	9,514.8	0.471	5,142	9,510.3	0.604	5,473	9,546.1
**Outpatient**	1,764	3,459.4	0.229	1,653	3,449.8	0.859	1,617	3,447.1
**Medical provider**	4,767	4,854.4	< 0.001	5,839	5,130.9	< 0.001	4,295	4,774.9
**Prescription drugs**	2,848	1,911.0	< 0.001	3,076	2,001.2	< 0.001	1,829	1,689.9
**Other**	2,530	9,510.6	< 0.001	3,656	9,498.9	0.352	3,330	9,493.8

Note: Based on 1,055 elderly community-dwelling Medicare beneficiaries aged 65 years or older, and followed for one year during 2001 through 2005. Expenditures were measured in the follow-up year and depression treatment was measured in year 1. *p*-values can be used to identify statistically differences in expenditures by depression treatment compared to no depression treatment based on t-tests.

^a^Due to small number of individuals who received only psychotherapy but no antidepressant treatment, we combined people having psychotherapy with and without antidepressant treatment into one group.

*p*: p-value; SD: Standard Deviation; Tx: Treatment; AD: Antidepressants; Psych Tx (with or w/o AD): Psychotherapy with or without antidepressants.

The percent increase or decrease associated with depression treatment categories (antidepressant use only and psychotherapy with and without antidepressants) compared to no depression treatment and 95% CI of these percentages for total, inpatient, outpatient, medical provider, prescription drugs, and other expenditures from separate linear regressions of log-transformed expenditures using OLS method are presented in Table 3. In the regressions after controlling for gender, race, age, marital status, metro status, education, poverty status, prescription drug coverage, health status, smoking status, BMI categories, year of observation and baseline log-transformed expenditures, total expenditures were significantly higher for those who used antidepressants only and psychotherapy with or without antidepressants compared to no depression treatment. Specifically, compared to no depression treatment, total expenditures were 20.2% (95% CI: 14.1-26.7%) greater for those with only antidepressants and 29.4% (95% CI: 18.8-41.0%) greater for those who used psychotherapy with or without antidepressants.

**Table 3:** Percent change, 95% confidence intervals of percent change depression treatment categories (Reference Group = No Depression Treatment) multiple linear regressions of log-transformed dollars (2005 $), medicare current beneficiary survey, 2000 – 2005

**Expenditures**	**Antidepressants only**			**Psych Tx (with or w/o AD)**		
	**% Change**	**95% CI**	**p-value**	**% Change**	**95% CI**	**p-value**
**Total**	20.2	[14.1 , 26.7]	<0.001	29.4	[18.8 , 41.0]	<0.001
**Inpatient**	31.4	[7.5 , 60.7]	0.008	56.9	[7.2 , 129.5]	0.021
**Outpatient**	11.1	[-2.4 , 26.3]	0.112	28.1	[ 0.0 , 64.2]	0.051
**Medical provider**	17.6	[12.1 , 23.5]	<0.001	28.6	[18.7 , 39.3]	<0.001
**Prescription drug**	26.8	[18.9 , 35.2]	<0.001	31.2	[20.2 , 43.2]	<0.001
**Other**	-6.8	[-20.6, 9.4]	0.389	13.5	[-15.2 , 51.9]	0.396

Note: Based on 1,055 community-dwelling elderly aged 65 years or older, and followed for one year during 2001 through 2005. Expenditures were measured in the follow-up year and depression treatment was measured in the baseline year.

Percent changes of healthcare expenditures for depression treatment categories were calculated from parameter estimates from separate regressions on total, inpatient, outpatient, medical provider, prescription drugs, and other expenditures which included dental, vision, and durable medical equipment. The models adjusted for other independent variables: predisposing (gender, race, age, year of observation), enabling (marital status, education, poverty status, prescription drug coverage), need (health status, functional status), personal health practices (smoking status, body mass index), healthcare use (baseline year log-transformed expenditures), and external environment (metro status) factors.

Tx: Treatment; AD: Antidepressants; Psych Tx(with or w/o AD): Psychotherapy with or without antidepressants.

Inpatient expenditures were 31.4% (95% CI: 7.5-60.7%) higher for those treated with antidepressants only and 56.9% (95% CI: 7.2-129.5%) higher for those treated with psychotherapy with or without antidepressants compared to no treatment. Medical provider expenditures were greater for those treated with antidepressants only, 17.6% (95% CI: 12.1-23.5%) and 28.6% (95% CI: 18.7-39.3%) higher for those treated with psychotherapy with or without antidepressants compared to no treatment. Similarly, prescription drug expenditures were 26.8% (95% CI: 18.9-35.2%) greater for those treated with antidepressants only and 31.2% (95% CI: 20.2-43.2%) higher for those treated with psychotherapy with or without antidepressants compared to those with no treatment.

## Discussion

Our study examined the association between depression treatment and short-term expenditures among the elderly with depression and chronic physical conditions such as arthritis, diabetes, respiratory diseases including asthma, chronic obstructive pulmonary disease, heart disease, hypertension, osteoporosis, and stroke. We found that among elderly Medicare beneficiaries with depression, those who received depression treatment had significantly greater total expenditures compared to those who did not receive any treatment for depression. These results are consistent with some studies in which treatment for depression was associated with greater expenditures [6,7]. Although, Simon and colleagues included all individuals who received some form of treatment for depression, their comparison was between collaborative care and usual care [6].

It is not surprising that depression treatment was associated with greater total healthcare expenditures in the short-term. It is possible to have chronic depression that can last for several years. There is some evidence of persistent depression in the general population and among those with persistent depression an overwhelming majority (87%) also had a chronic condition. Among veterans with diabetes, heart disease, and hypertension, 5.6% were diagnosed with persistent depression [27] The STAR*D (Sequenced Treatment Alternatives to Relieve Depression) trial, in which many individuals had comorbid chronic health conditions, revealed that relapse rates could be as high as 50% even among individuals who were in remission in a stepped treatment approach [28,29]. Even the ENRICHD (Enhancing Recovery In Coronary Heart Disease) trial which focused on nonpharmacologic treatment of depression, in patients with myocardial infarction as well as depression and/or low perceived social support, showed that psychological outcomes (reduced score on the Beck Depression Inventory scale) improved at six months but did not last up to 30 months [15].

Greater short-term expenditures among those with depression treatment compared to those without any depression treatment could be due to early discontinuation of medications. In one study of a large private health insurance database, total healthcare expenditures for individuals, with equal distribution of comorbid conditions, who used antidepressants for 3 months were significantly lower than expenditures of those who discontinued the medications early [30]. Using healthcare claims from 30 health plans, Katon and colleagues analyzed the relationship between adherence to depression treatment and total medical charges among specific chronic conditions. This study found that adherence to depression treatment reduced total medical charges [31]. However, our study could not measure adherence to depression treatment due to unavailability of date of pharmacy claims. Our study included elderly with co-occurring depression any number of chronic conditions. The presence of chronic diseases may be associated with or induce biological changes that render otherwise useful treatments for depression ineffective [29]. Indeed, the SADHART (Sertraline Antidepressant Heart Attack Randomized Trial) trial compared antidepressant treatment against placebo in depressed patients with acute myocardial infarction or unstable angina and did not report any significant difference in the incidence of severe cardiac events (death, myocardial infarction, congestive heart failure, stroke, and recurrent angina) between the antidepressant group and those who received a placebo [13]. Among individuals with chronic conditions, even when depression treatment is successful the ENRICHD trial revealed that depression treatment did not decrease the risk of event free survival after an MI [14]. In a confirmatory analysis of previously unanalyzed cohort, the STAR*D trial found that individuals with medical conditions and major depressive disorder were a particularly disadvantaged group with significant challenges to clinical management [32]. Thus, it is possible that among individuals with co-occurring depression and chronic conditions, the relationship between depression treatment and expenditures may be complex and may not reveal a reduction in expenditures in the short-term. Our results, therefore, suggest the need for examining the association between depression treatment and healthcare expenditures using a longer period of follow-up.

For each type of medical care services, we found a statistically significant relationship between depression treatment and healthcare expenditures except for the “outpatient” and “other” categories. Further research is needed as to whether adequate depression treatment may reduce inpatient expenditures because studies in the veterans administration and elsewhere have documented that among individuals with depression, those who had adequate duration of antidepressants were less likely to be hospitalized than those who did not have adequate duration [33]. Similarly, Katon and colleagues reported that in individuals with poorly controlled diabetes, coronary heart disease, or both and coexisting depression, collaborative care produced positive outcomes for chronic care and this may indirectly lead to reduced inpatient care for depression [5]. In this study we could not assesses adequate depression treatment due to lack of information on prescription dates or whether collaborative care was provided.

We observed significantly higher expenditures in prescription drug expenditures for those who received depression treatment compared to those who did not. This may be due to the fact that prescription drug expenditures in our study may also include payments for antidepressants as the main modality for treatment in our sample. One could also speculate that those who use antidepressants may also be using other medications for conditions that are comorbid with depression (e.g., anxiety).

The findings from the current study have to be considered in the context of its strengths and limitations. The study had many strengths such as longitudinal study design, nationally representative sample of the elderly, inclusion of psychotherapy use with or without antidepressants, comprehensive list of independent variables, and complete utilization that included both Medicare and non-Medicare expenditures. We also derived depression from diagnosis codes of inpatient and outpatient claims. Limitations of the study are recall bias because some data were queried only in the survey. For example, antidepressant use was based on self-reports because prescription drug was not covered by Medicare during our study period. However to minimize recall errors, the MCBS had put in place many steps such as requesting the respondents to bring their prescription bottles, facilitating recording of all their healthcare events, and the list of medications used in the previous rounds by the respondent to the interviewers. In addition, we did not measure discontinuation of depression treatment which may be associated with greater short-term healthcare expenditures. Although depression diagnosis was derived from claims and may indicate clinical need; by using diagnosed depression, we may have excluded individuals for whom depression diagnoses was not coded. Our study was retrospective and used observational data, therefore may have unmeasured confounding factors. For example, individuals in depression treatment groups may systematically differ from those without treatment on unobserved and unmeasured characteristics such as preferences and motivation that could explain some of the relationship between depression treatment and healthcare expenditures. Finally, due to the lack of individuals in our study period who reported being on both an antidepressant and were involved in psychotherapeutic treatment, we were not able to fully evaluate costs related to such combined treatment.

## Conclusion

Despite these limitations our study contributed to the nascent literature on the relationship between depression treatment and healthcare expenditures in individuals with chronic physical conditions. Among individuals treated with either antidepressants or psychotherapy, depression treatment was associated with greater short-term expenditures. For example, individuals having antidepressants use only had 20% increase in total expenditures compared to those with no treatment; while individuals having psychotherapy with or without antidepressants had a 31% increase. Similarly, medical provider expenditures were 17.6% higher for those treated with antidepressants only compared to those with no treatment for depression. Our study findings highlight the need for future studies that include longer follow-up period and the need to control for treatment-resistant depression.

## Competing interests

The authors have no conflicts of interests or competing interests to declare.

## Authors’ contributions

US designed the study. NS produced the first draft of the manuscript. US and CS participated in statistical analysis. PF contributed to data purchase and editing of the manuscript. CS, PF, NS, and US participated in successive drafts of the manuscript. All authors read and approved the final manuscript.

## Authors’ information

At the time the study was conducted and originally submitted, Neel Shah was a PhD candidate at West Virginia University.

## References

[B1] Egede LE Zheng D Simpson K Comorbid depression is associated with increased health care use and expenditures in individuals with diabetes Diabetes Care 2002 12 464 470 10.2337/diacare.25.3.464 11874931

[B2] Simon GE Manning WG Katzelnick DJ Pearson SD Henk HJ Helstad CS Cost-effectiveness of systematic depression treatment for high utilizers of general medical care Arch Gen Psychiatry 2001 12 181 187 10.1001/archpsyc.58.2.181 11177120

[B3] Simon GE Katon WJ Lin EH Rutter C Manning WG Von Korff M Ciechanowski P Ludman EJ Young BA Cost-effectiveness of systematic depression treatment among people with diabetes mellitus Arch Gen Psychiatry 2007 12 65 72 10.1001/archpsyc.64.1.65 17199056

[B4] Tiwari A Rajan M Miller D Pogach L Olfson M Sambamoorthi U Guideline-consistent antidepressant treatment patterns among veterans with diabetes and major depressive disorder Psychiatr Serv 2008 12 1139 1147 10.1176/appi.ps.59.10.1139 18832499

[B5] Katon WJ Lin EH Von Korff M Ciechanowski P Ludman EJ Young B Peterson D Rutter CM McGregor M McCulloch D Collaborative care for patients with depression and chronic illnesses N Engl J Med 2010 12 2611 2620 10.1056/NEJMoa1003955 21190455 PMC3312811

[B6] Simon GE Katon WJ VonKorff M Unützer J Lin EH Walker EA Bush T Rutter C Ludman E Cost-effectiveness of a collaborative care program for primary care patients with persistent depression Am J Psychiatry 2001 12 1638 1644 10.1176/appi.ajp.158.10.1638 11578996

[B7] Gilbody S Bower P Fletcher J Richards D Sutton AJ Collaborative care for depression: a cumulative meta-analysis and review of longer-term outcomes Arch Intern Med 2006 12 2314 2321 10.1001/archinte.166.21.2314 17130383

[B8] Nutting PA Rost K Smith J Werner JJ Elliot C Competing demands from physical problems: effect on initiating and completing depression care over 6 months Arch Fam Med 2000 12 1059 1064 10.1001/archfami.9.10.1059 11115208

[B9] Rost K Nutting P Smith J Coyne JC Cooper-Patrick L Rubenstein L The role of competing demands in the treatment provided primary care patients with major depression Arch Fam Med 2000 12 150 154 10.1001/archfami.9.2.150 10693732

[B10] Klinkman MS Competing demands in psychosocial care. A model for the identification and treatment of depressive disorders in primary care Gen Hosp Psychiatry 1997 12 98 111 10.1016/S0163-8343(96)00145-4 9097064

[B11] Pan A Sun Q Okereke OI Rexrode KM Rubin RR Lucas M Willett WC Manson JE Hu FB Use of antidepressant medication and risk of type 2 diabetes: results from three cohorts of US adults Diabetologia 2011 12 63 72 21811871 10.1007/s00125-011-2268-4 PMC3229672

[B12] Kivimäki M Hamer M Batty GD Geddes JR Tabak AG Pentti J Virtanen M Vahtera J Antidepressant medication use, weight gain, and risk of type 2 diabetes: a population-based study Diabetes Care 2010 12 2611 2616 10.2337/dc10-1187 20823343 PMC2992199

[B13] Glassman AH O’Connor CM Califf RM Swedberg K Schwartz P Bigger JT Jr Krishnan KR van Zyl LT Swenson JR Finkel MS Landau C Shapiro PA Pepine CJ Mardekian J Harrison WM Barton D Mclvor M Sertraline Antidepressant Heart Attack Randomized Trial (SADHEART) Group. Sertraline treatment of major depression in patients with acute MI or unstable angina JAMA 2002 12 701 709 10.1001/jama.288.6.701 12169073

[B14] Joynt KE O’Connor CM Lessons from SADHART, ENRICHD, and other trials Psychosom Med 2005 12 Suppl 1 S63 S66 15953805 10.1097/01.psy.0000163454.25036.fc

[B15] Andersen RM Revisiting the behavioral model and access to medical care: does it matter? J Health Soc Behav 1995 12 1 10 10.2307/2137284 7738325

[B16] Rayner L Price A Evans A Valsraj K Higginson IJ Hotopf M Antidepressants for depression in physically ill people Cochrane Database Syst Rev 2010 12 CD007503 10.1002/14651858.CD007503.pub2 PMC12279289 20238354

[B17] Centers for Medicare and Medicaid Services Medicare Current Beneficiary Survey (MCBS) related files Available at: https://www.cms.gov/MCBS. Accessed 07/20, 2011

[B18] 2011 Quick Reference Guide (HEDIS/QARR MEASURES) Available at: http://www.healthfirstny.org/pdf/Provider-Materials/Provider-Quick-Reference-Guide-HEDIS-QARR-Measures-2011_NY.pdf. Accessed 07/07, 2011

[B19] Wei W Sambamoorthi U Olfson M Walkup JT Crystal S Use of psychotherapy for depression in older adults Am J Psychiatry 2005 12 711 717 10.1176/appi.ajp.162.4.711 15800143 PMC2921918

[B20] Crystal S Sambamoorthi U Walkup JT Akincigil A Diagnosis and treatment of depression in the elderly medicare population: predictors, disparities, and trends J Am Geriatr Soc 2003 12 1718 1728 10.1046/j.1532-5415.2003.51555.x 14687349 PMC2486833

[B21] Sambamoorthi U Olfson M Walkup JT Crystal S Diffusion of new generation antidepressant treatment among elderly diagnosed with depression Med Care 2003 12 180 194 10.1097/00005650-200301000-00019 12544554

[B22] Sambamoorthi U Shen C Findley P Frayne S Banerjea R Sambamoorthi U Shen C Findley P Frayne S Banerjea R Depression treatment patterns among women veterans with cardiovascular conditions or diabetes World Psychiatry 2010 12 177 182 20975865 10.1002/j.2051-5545.2010.tb00306.x PMC2948721

[B23] Bureau of Labor Statistics Consumer Price Index Detailed Report Tables Available at: http://www.bls.gov/cpi/cpid05av.pdf. Accessed 07/20, 2011

[B24] Centers for Disease Control and Prevention (CDC) BMI Classification [online] Available at: http://www.cdc.gov/healthyweight/assessing/bmi/adult_bmi/index.html. Accessed 10/03, 2011

[B25] Monheit AC Persistence in health expenditures in the short run: prevalence and consequences Med Care 2003 12 7 Suppl III53 III64 12865727 10.1097/01.MLR.0000076046.46152.EF

[B26] Halvorsen R Palmquist R The interpretation of dummy variables in Semilogarithmic equations Am Econ Rev 1980 12 474 475

[B27] Findley P Shen C Sambamoorthi U Multimorbidity and persistent depression among veterans with diabetes, heart disease, and hypertension Health Soc Work 2011 12 109 119 10.1093/hsw/36.2.109 21661300

[B28] Masand P Narasimhan M Major depressive disorder and medical illness: STAR*D outcomes Curr Psychiatry Rep 2006 12 165 166 10.1007/s11920-006-0017-z 19817064

[B29] Rush AJ Trivedi MH Wisniewski SR Nierenberg AA Stewart JW Warden D Niederehe G Thase ME Lavori PW Lebowitz BD McGrath PJ Rosenbaum JF Sackeim HA Kupfer DJ Luther J Fava M Acute and longer-term outcomes in depressed outpatients requiring one or several treatment steps: a STAR*D report Am J Psychiatry 2006 12 1905 1917 10.1176/appi.ajp.163.11.1905 17074942

[B30] Thompson D Buesching D Gregor KJ Patterns of antidepressant use and their relation to costs of care Am J Manag Care 1996 12 1239 1246

[B31] Katon W Cantrell CR Sokol MC Chiao E Gdovin JM Impact of antidepressant drug adherence on comorbid medication use and resource utilization Arch Intern Med 2005 12 21 2497 2503 10.1001/archinte.165.21.2497 16314547

[B32] Yates WR Mitchell J Rush AJ Trivedi MH Wisniewski SR Warden D Hauger RB Fava M Gaynes BN Husain MM Bryan C Clinical features of depressed outpatients with and without co-occurring general medical conditions in STAR*D Gen Hosp Psychiatry 2004 12 421 429 10.1016/j.genhosppsych.2004.06.008 15567207

[B33] Charbonneau A Rosen AK Owen RR Spiro A 3rd Ash AS Miller DR Kazis L Kader B Cunningham F Berlowitz DR Monitoring depression care: in search of an accurate quality indicator Med Care 2004 12 522 531 10.1097/01.mlr.0000127999.89246.a6 15167320

